# Highly Conductive Carbon Fiber Reinforced Concrete for Icing Prevention and Curing

**DOI:** 10.3390/ma9040281

**Published:** 2016-04-12

**Authors:** Oscar Galao, Luis Bañón, Francisco Javier Baeza, Jesús Carmona, Pedro Garcés

**Affiliations:** Civil Engineering Department, University of Alicante, Ctra. San Vicente s/n, San Vicente del Raspeig 03690, Spain; oscar.galao@ua.es (O.G.); lbanon@ua.es (L.B.); fj.baeza@ua.es (F.J.B.); jcarmona@ua.es (J.C.)

**Keywords:** conductive concrete, deicing, heating, carbon fibers, multifunctional composites

## Abstract

This paper aims to study the feasibility of highly conductive carbon fiber reinforced concrete (CFRC) as a self-heating material for ice formation prevention and curing in pavements. Tests were carried out in lab ambient conditions at different fixed voltages and then introduced in a freezer at −15 °C. The specimens inside the freezer were exposed to different fixed voltages when reaching +5 °C for prevention of icing and when reaching the temperature inside the freezer, *i.e.*, −15 °C, for curing of icing. Results show that this concrete could act as a heating element in pavements with risk of ice formation, consuming a reasonable amount of energy for both anti-icing (prevention) and deicing (curing), which could turn into an environmentally friendly and cost-effective deicing method.

## 1. Introduction

In cement composites, multifunctionality consists of taking advantage of the structural material itself to develop nonstructural functions, without the need of any type of external device. That can be achieved by combining a cementitious material with different additions that provide the resulting material with a new range of applications [1,2,3,4,5,6,7,8,9,10], keeping or even improving its structural characteristics [11,12,13,14,15,16,17]. Thus, cost is reduced, design is simplified, and the use of embedded devices is minimized. Functional properties include: anode for electrochemical chloride extraction [2,3], electromagnetic wave shielding [5], strain/stress sensor [6,7,8], dynamic monitoring and damage detection [8,10,18], temperature sensor [19], heating and thermal control [20,21,22,23,24,25,26], among others. With its application as an ice controller on different transportation infrastructures (e.g., highways, interchanges, bridges, airport runways), safety for the drivers would be increased, while not compromising the durability of the structures with the use of sprayed substances that can damage them.

Carbonaceous materials have a high thermal conductivity—although not as high as that of metals, plus a low coefficient of thermal expansion lower than that of metals—and are highly resistant to corrosion [13,14,15], which makes them good candidates for thermal applications in multifunctional cementitious composites such as the heating of buildings or pavement deicing. Carbon fiber is one of these materials which, used as an addition to concrete, can transform the original high resistivity to yield an electrically conductive cement composite, *i.e.*, carbon fiber reinforced concrete (CFRC) [1].

To achieve good heating results, a high current would be needed if the electrical resistivity is too low, while a high voltage needs to be applied if the electrical resistivity is too high (with very low current values) [22]. So far, the study of self-heating concrete has been mainly based on composites with medium electrical resistivity. In fact, other researchers have investigated the deicing performance of CFRC with an electrical resistivity of only 10 Ω·cm at 30 °C [27] with promising results.

Many methods and techniques have been investigated for pavement anti-icing and deicing as snow and ice on roads cause enormous losses of human lives, infrastructure, and materials [1,20,28,29,30,31,32,33,34,35,36,37,38,39,40,41,42]. The mechanical snow removal technique is the most used but it is an intensive, expensive and time-consuming labor. Furthermore, not all the snow is completely cleaned from the driveway by the snowplows, leaving a small layer to be eliminated. Many of the methods currently used to remove ice from roads are based on the use of chemicals. Most of them are severely harmful to both reinforced concrete and steel structures, viaducts, tunnels, airport runways, as well as to the environment [43,44,45,46,47,48].

A number of researchers have analyzed the feasibility of using conductive multifunctional concrete, with different additions, for pavement deicing [29,49,50,51,52]. Since the late 1990s, steel fibers, steel shavings, carbon fibers, and graphite products have been added into concrete as conductive materials to greatly improve the electrical conductivity. Some drawbacks of using steel shavings in the mixtures were noticed, and thus carbon products were used to replace the steel shavings in the conductive concrete mix design.

A conductive concrete deck using carbon products of different particle sizes was implemented for deicing on a 36-meter-long and 8.5-meter-wide highway bridge in Roca, Nebraska [50]. The deicing system worked well in four major snowstorms in the winter of 2003, delivering an average power density of 452 W/m^2^ to melt snow and ice. A method of deicing with carbon fiber heating wires (CFHWs) embedded inside concrete slabs has also been verified, showing that, with an input power of 1134 W/m^2^, the temperature on the slab surface rises from −25 °C to above 0 °C in 2.5 h at an approximate rate of 0.17 °C/min [52].

A previous research [26] studied the heating caused by the flow of electrical current through a cement paste containing carbonaceous materials such as nanofibers, nanotubes, carbon fiber powder and graphite powder, which reduce the electrical resistance of the resulting composite. An experimental study and a mathematical model were developed from it. That research showed the best results thus far in cement composites searching for high electrical conductivity.

With the purpose of studying the thermal function, this research is focused on the heating effect produced by the electrical current passing through specimens made of concrete with the addition of carbon fibers, which transform an ordinary concrete into a conductive concrete. AC and DC power sources at different fixed voltages were used to study two different actions in this research: anti-icing (prevention) and deicing (curing). For prevention, specimens initially at room temperature were introduced into the freezer with a temperature of −15 °C, until they reached approximately +5 °C. For curing, specimens were kept inside the same freezer for 24 h, reaching the internal ambient temperature, *i.e.*, −15 °C.

## 2. Experimental Program and Materials

### 2.1. Materials and Sample Fabrication

Concrete specimens of 30 × 30 × 2 cm^3^ were fabricated with the following materials: Portland cement CEM-I 52.5 R according to European Standard [53]; tap water; standard silica sand (EN 196-1), round river gravel 3–6 mm; silica fume (SF) EMS968; superplasticizer; oxidized carbon fibers (CF), whose properties are included in Table 1. Concrete dosage, based on previous research [54], is shown in Table 2. Water/cement ratio was 0.50, CF content was 2.0% by weight of cement and SF/cement content was 10% by weight of cement. To improve fiber dispersion and composite workability (main drawbacks that arise when mixing CF with cement composite [1,55,56,57,58]), the following measures were taken:

- 2.1% by weight of cement polycarboxylate-based superplasticizer was added to the mix.
- In order to improve dispersion, CF oxidation treatment was conducted by placing the fibers in a furnace at 400 °C with an air flow of 10 mL/min for 4 h [12,59].
- The oxidized CF material was poured in water and then ultrasound treatment was applied for 10 min with an ultrasound device model Hielschier UP200S [8].

According to the electrical resistivity obtained in the tests a relatively good CF dispersion can be assumed [1].

**Figure 1:**
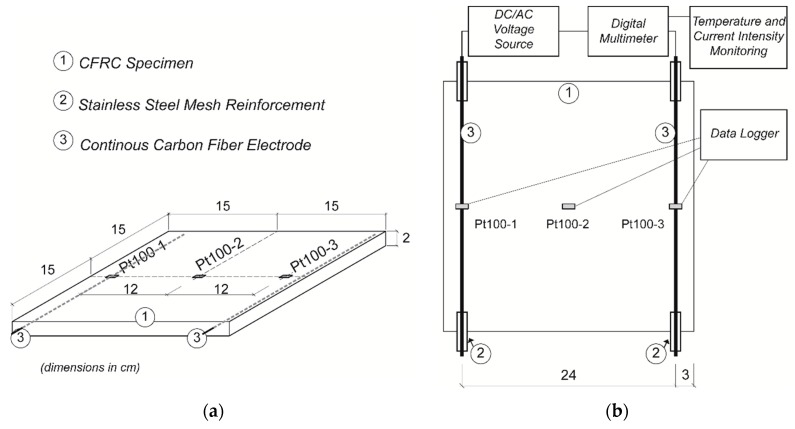
(**a**) Specimen geometry and location of temperature sensors, Pt100; (**b**) Experimental setup for self-heating test.

The electrodes were fabricated with continuous polyacrylonitrile (PAN) carbon fibers, which were wrapped together with 0.9-mm-thick stainless steel mesh at the end parts in order to prevent the fibers from breaking when being handled. Their position is shown in Figure 1.

**Figure 2:**
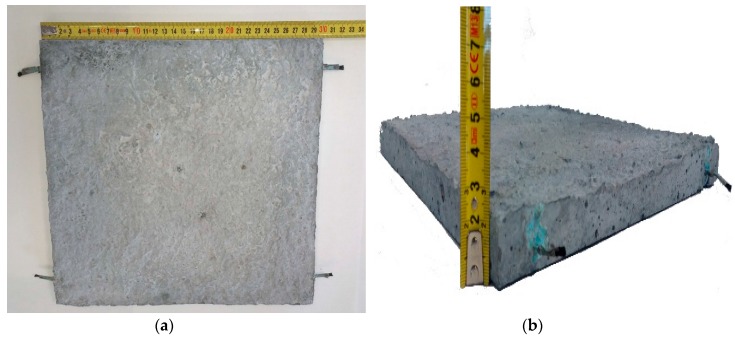
Specimen appearance after fabrication: (**a**) top view; (**b**) thickness.

The mixture was poured into stainless steel molds with the electrodes already positioned. After 24 h curing in humidity chamber at 20 °C, 95% relative humidity, the specimens were demolded and kept in the same conditions for 28 days. Specimen A was 300 mm × 300 mm × 21.8 mm and specimen B was 300 mm × 300 mm × 20.7 mm. Figure 2 shows the specimens appearance after fabrication: (a) top view; (b) thickness.

**Figure 3:**
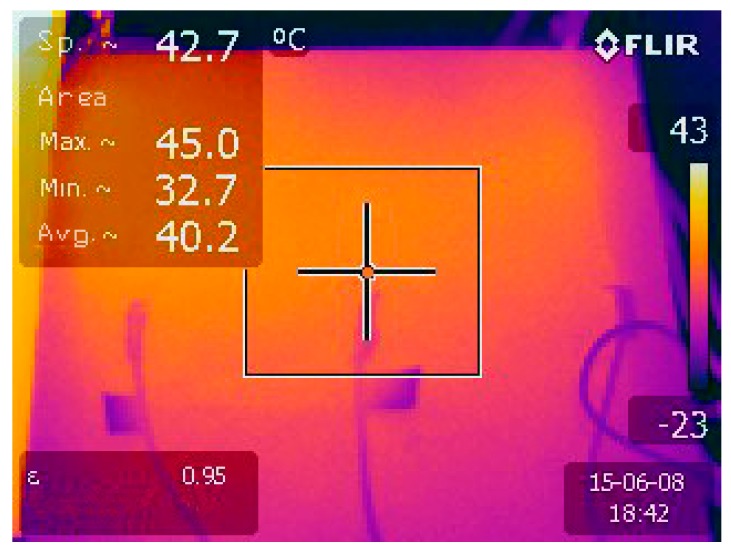
Specimen temperature controlled with an IR camera.

Changes in the surface temperature of the specimens were continuously registered by RTD temperature sensors type Pt100, distributed as shown in Figure 1 and connected to a data logger. A FLIR E30 thermographic camera was also used to control the temperature distribution along the specimen’s surface, as shown in Figure 3.

In direct current, fixed voltage was applied with a digital direct power source. In alternating current (50 Hz), fixed voltage was applied using an alternating power source F5V. In both cases, electrical current was measured with digital multimeters Keithley 2002. A general scheme is shown in Figure 1.

As shown in Figure 1, three Pt100 sensors were attached to each specimen in order to control its temperature. Another Pt100 sensor was placed at the top center of the freezer to control environment temperature.

All power sources were kept inside a security chamber for safety concerns during tests. Moreover, all cables used were shielded for noise signal reduction and for security concerns too. No thermal insulator was utilized; otherwise, the results obtained would have shown a much higher efficiency.

### 2.2. Laboratory Conditions Tests (L.C.)

Before testing, the specimens were kept in laboratory conditions: approximately 21 °C and 55% relative humidity for 14 days. Testing voltages were fixed to 10, 20 and 25 V. Different specimens were used for DC tests and for AC tests. All specimens were kept in the same chamber in laboratory conditions until the end of tests, for safety concerns.

### 2.3. Prevention and Curing Tests

After the tests in laboratory conditions, the specimens were exposed to the same alternating or direct currents for prevention and curing tests. Testing voltages were in this case fixed to 20 and 25 V. A Liebherr freezer was utilized whose inner dimensions were 1.45 m × 0.50 m × 0.65 m. The freezer average ambient temperature was −15 °C.

In prevention tests, specimens at room temperature were put into the freezer. When they reached approximately +5 °C, both power sources (AC and DC) were connected at a fixed voltage. After steady temperature was reached, power sources were turned off.

In curing tests, specimens were kept inside the freezer for 24 h, reaching the inside temperature, *i.e.*, −15 °C. Then both power sources (AC and DC) were connected at a fixed voltage. After about 4 h, power sources were turned off. Temperatures were monitored until the specimens reached again the same temperature as the freezer inside temperature in both prevention and curing tests.

## 3. Results and Discussion

Table 3 summarizes the results obtained in the tests, including: test type laboratory conditions (L.C.), prevention (Prev.) or curing (Cur.); current type used—AC or DC—and fixed voltage, in V; monitored average current, in A, during the test and its calculated standard deviation (%SD), in percentage; time with the current on, in hours; calculated energy consumption, in J; average power, in W/m^2^; average electrical resistivity, in Ω·cm; initial environment temperature (Tenv), in °C; initial specimen average temperature (T_concrete), in °C; variation between the initial and final specimen temperature (ΔT_concrete), in °C; variation in temperature between the final average specimen temperature and the initial environment temperature (ΔT_concrete-env), in °C; and average power per unit °C needed to increase specimen temperature over environment temperature, in W/(m^2^·°C).

**Figure 4:**
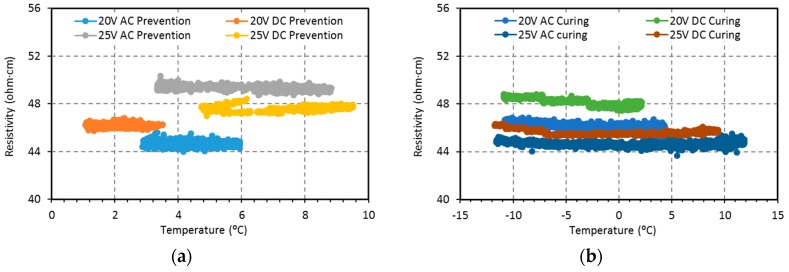
Variation of electrical resistivity with temperature: (**a**) 20 V and 25 V, AC and DC, prevention test; (**b**) 20 V and 25 V, AC and DC, curing test.

It should be highlighted that the electrical conductivity obtained in the fabricated concrete is about two orders of magnitude larger than the ones reported by some previous studies [24], although it is smaller than others [27]. In accordance with the high conductivity of the specimens, the electrical current monitored increased with the fixed voltage. The electrical resistance (*Z*) of a material can be expressed as *Z* = Ψ × *L*/*S*, where Ψ is the average electrical resistivity of a cross-sectional area; *L* is the spacing between electrodes; and *S* is the area of a cross-section parallel to the electrodes. According to Table 3 and Ohm’s Law, the average electrical resistance—or impedance in AC, considering no phase lag—for all the tests is 17.9 ± 0.9 Ω; moreover, no major polarization phenomena were observed. Figure 4 shows the calculated electrical resistivity *vs.* the specimen’s average temperature monitored every 5 s, for 20 V and 25 V, AC and DC, prevention and curing tests. It can be observed that the electrical resistivity kept its steady state through all the experiment time with negligible variation. According to other studies, a decrease in resistivity with increasing temperature should have been expected [19]. All this is relevant in order to achieve system stability.

For each test condition, as expected, the higher the voltage applied, the higher the temperature the specimens reached, for both AC and DC tests. The average power, in W/m^2^, was similar for tests with the same current type and fixed voltage. Also very similar were the results obtained with AC and DC, *i.e.*, increment of temperature, energy consumption, electrical current, *etc*.

### 3.1. Laboratory Conditions Tests

Specimens in laboratory conditions of 25 °C and 50% relative humidity, approximately, were tested with fixed voltages of 10 V, 20 V and 25 V. After the specimen temperature reached a stable value, the power was turned off. Temperatures were monitored until environment and specimen temperatures matched again.

**Figure 5:**
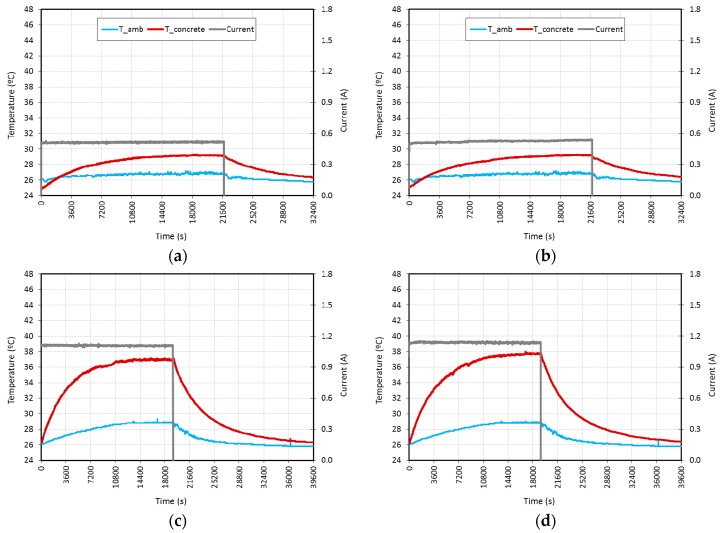

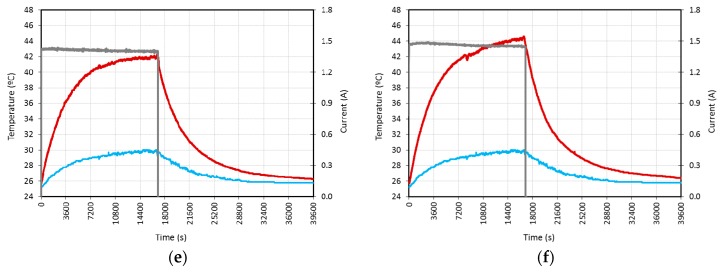
L.C. tests performed at different fixed voltages: (**a**) 10 V AC; (**b**) 10 V DC; (**c**) 20 V AC; (**d**) 20 V DC; (**e**) 25 V AC; (**f**) 25 V DC.

Figure 5 shows the environment temperature and the average specimen temperature (left axis, in °C) and the electrical current (right axis, in A), for AC and DC, at 10 V, 20 V and 25 V fixed voltages *vs.* time (horizontal axis, in s), monitored during the tests. The environment temperature sensor was placed about 15 cm above the specimen, so the influence of heat produced by the specimens can be observed in its results.

The temperature increment was similar for both current types, approximately of 3 °C at 10 V, 11 °C at 20 V and 17 °C at 25 V, although tests at 25 V show that a higher temperature could have been obtained as steady state was not reached—the temperature could not stay constant—after almost 5 h; at that moment, power was turned off.

The average electrical resistivity, in Ω·cm, measured in the AC tests was 52.8 at 10 V, 49.1 at 20 V and 48.2 at 25 V, whereas in the DC tests it was 49.7 at 10 V, 45.3 at 20 V and 45.7 at 25 V. These values are in accordance with previous research in which, for a CFRC with similar dosage, 20.14% ± 6% Ω·cm was obtained in DC measures with 4 × 4 × 16 cm³ specimens [54]. The differences in conductivity could be explained by the SF addition, which has been reported in several studies [1,55].

The highest variation of the electrical current observed during tests was for 10 V DC L.C. (2.1%) and 25 V AC L.C. (1.0%), although in the rest of the tests that variation was smaller than 1%. The former variations are relatively low, and thus the energy consumed can be calculated according to Q = P × t, where *P* is the power output (P = V × I) and *t* is the time with the power source on. As shown in Table 3, the average power, in W/m^2^, of the AC tests was 71.6 at 10 V, 307.7 at 20 V and 489.1 at 25 V, whereas that of the DC tests was 72.6 at 10 V, 315.7 at 20 V and 506.5 at 25 V. The surface considered for this calculation was 30 cm × 24 cm—the length between electrodes—instead of 30 cm × 30 cm.

**Figure 6:**
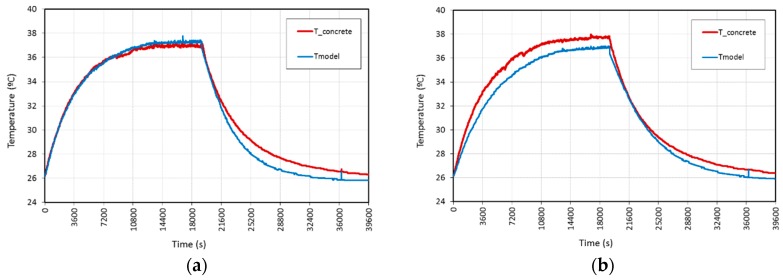
Temperature comparison between experimental results and mathematical model for L.C. tests at different types of voltage: (**a**) 20 V AC; (**b**) 20 V DC.

According to the stability of the electrical variables current and, consequently, the power and resistance, as the voltage was fixed and there was high conductivity, the same mathematical modelization as in [26], based on Newton’s Law of Cooling, was implemented with very good results. As an example, Figure 6 shows the correlation for 20 V AC and DC laboratory conditions tests. The equations describing the model were Equation (1) for the heating stage, Equation (2) for the steady stage and Equation (3) for the cooling stage.

(1) $$T=T_{r}+\frac{P}{hA}\left[1-e^{-\frac{hA}{mc_{p}}\left(t-t_{1}\right)}\right]$$

(2) $$T=T_{r}+\frac{P}{hA}$$

(3) $$T=T_{r}+\left(T_{off}-T_{r}\right)e^{-\frac{hA}{mc_{p}}\left(t-t_{off}\right)}$$

where *T* (°C) is the average temperature of the specimen, *T_r_* (°C) is the room temperature, *P* (W) is the applied power, *h* (W/(m^2^·°C)) is the heat transfer coefficient (a parameter that measures the energy transfer rate to the environment), *A* (m^2^) is the surface of the sample exposed, *m* (kg) is the mass of the sample, *c_p_* (J/kg·°C) is the specific heat of the material of the sample, *t* (s) is the time, *t*_1_ (s) is the time elapsed in steady state, *T*_off_ is the temperature of the specimen when the electrical power is disconnected, and *t*_off_ (s) is the lapsed time at disconnection.

### 3.2. Prevention Tests

**Figure 7:**
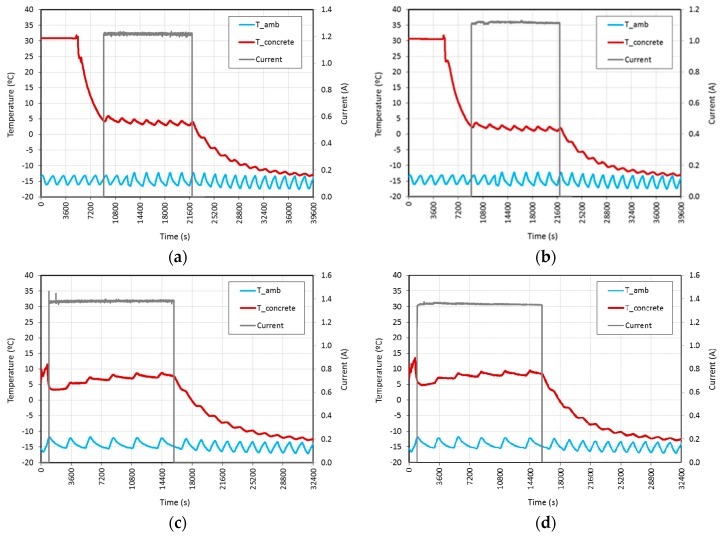
Prevention tests performed at fixed voltages of: (**a**) 20 V AC; (**b**) 20 V DC; (**c**) 25 V AC; (**d**) 25 V DC.

In these tests, the specimens at an environment temperature of approximately 25 °C were placed into the freezer at −15 °C. When the temperature of the specimens reached about +5 °C the power was turned on for both AC and DC. As shown in Table 3 and Figure 7, the results obtained were quite similar in both AC and DC tests. With a voltage of 20 V, both AC and DC were able to maintain the temperature of the specimen at above 0 °C throughout the whole test duration. With 25 V, also with the AC and DC current types, the temperature of both specimens increased more than 3 °C from the initial values. The average current was between 1.1 and 1.4 A and the average power, in W/m^2^, was 338.7 at 20 V and 479.3 at 25 V for AC tests, and 310.3 at 20 V and 470.2 at 25 V for DC tests. The periodic tendency observed in the ambient temperature is due to freezer regulation hysteresis. The same tendency with little or no inertia was observed in the temperature of the specimens monitored.

The variation of temperature between the initial environment temperature and the final temperature of the specimens is shown in Table 3. This parameter is remarkably higher in prevention (and curing) tests than in laboratory conditions tests, at the same voltage. This fact could be initially attributed to differences in air heat transfer coefficient values (h) caused by different air temperatures of +25 °C *vs.* −15 °C, and also because of different boundary conditions affecting L.C. tests, carried out in a fume hood instead of in the freezer, allowing a higher heat loss through the surface due to the air convection effect. The maximum value, 24.5 °C, was obtained during 25 V DC curing tests.

### 3.3. Curing Tests

In these tests, specimens were previously kept inside the freezer for 24 h, so they could reach the inside temperature, *i.e.*, −15 °C. Then, both power sources (AC and DC) were connected at a fixed voltage. After approximately 4 h, power sources were turned off. Temperatures were monitored until the specimens again reached the same temperature as the freezer ambient.

**Figure 8:**
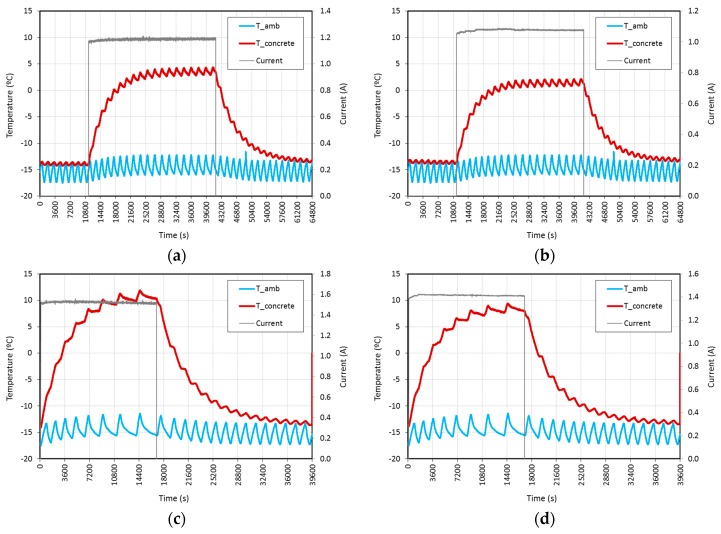
Curing tests performed at fixed voltages of: (**a**) 20 V AC; (**b**) 20 V DC; (**c**) 25 V AC; (**d**) 25 V DC.

As shown in Table 3 and Figure 8, the results obtained were quite similar for specimens A and B. With a voltage of 20 V both AC and DC were able to increase the temperature of the specimen above 0 °C in about 2 h for AC and 2.5 h for DC tests, and in less than 1 h for AC and DC tests performed at 25 V. The average current was between 1.1 and 1.5 A and the average power, in W/m^2^, of specimen A (AC tests) was 329.4 at 20 V and 526.0 at 25 V, whereas that of specimen B (DC tests) was 298.9 at 20 V and 489.2 at 25 V. As previously indicated in prevention tests, the periodic tendency observed in the ambient temperature is due to freezer regulation hysteresis. The same tendency with little or no inertia was observed in the temperature of the specimens monitored.

The average power (W/m^2^) was similar for the majority of the tests carried out in laboratory conditions, prevention and curing with fixed voltages of 20 V and 25 V. Obviously, a higher amount of energy is required to increase the ice temperature above 0 °C (water phase transition). Also, the final temperature of the specimens is very similar when comparing the same current conditions for prevention and curing tests: 4.5–4.3 for 20 V AC; 2.5–2.2 for 20 V DC; 7.9–11.9 for 25 V AC; 9.5–9.4 for 25 V DC. This is in accordance with the high stability of the system.

The variations in temperature between the initial environment temperature and the final temperature of the specimens are shown in Table 3. As previously stated, this parameter is remarkably higher in prevention and curing tests than in laboratory conditions tests, especially taking into account the water phase transition energy required. The maximum, 26.9 °C, was obtained with 25 V AC curing tests.

After the tests shown in this paper, the specimens were kept 28 days in a humidity chamber. The electrical resistance was periodically measured for two months at approximately 25 °C with a FLUKE multimeter, obtaining an average value of 20.7 ± 0.4 Ω. This result, very similar to those obtained during the tests, shows the stability of the electrical properties of the specimens.

## 4. Conclusions

After all results have been discussed, the following conclusions can be drawn:

- Both AC and DC are feasible for increasing the specimen temperature.
- Fixed voltages of 20 V or 25 V allowed specimens both to prevent freezing and to increase their temperature above 0 °C in an initial environment of −15 °C curing process. Consequently, a fixed voltage of 20 V could be enough to make the specimen work as a heating element with a very reasonable amount of energy consumption.
- The mathematical model published in [26] was checked in laboratory conditions tests, showing a very good correlation, and therefore being applicable for this type of highly conductive concrete in the previously mentioned conditions.

## Figures and Tables

**Table 1:** Carbon fiber properties.

Property	Value and Unit
Diameter	7.2 µm
Length	1/3 in (8.5 mm)
Carbon content	95%
Tensile strength	3800 MPa
Elastic modulus	242 GPa
Resistivity	1.52 × 10^−3^ Ω·cm
Density	1.81 g/cm^3^

**Table 2:** Concrete dosage (g).

Cement	5400
Water	2700
Sand	4050
Gravel	6075
Silica Fume	540
Superplasticizer	113.4
Carbon Fibers	108

**Table 3:** Summary of the results obtained in all tests.

Test Type ^1^	Current Type and Voltage (V)	Avg. ^2^ Curr. (A)	% SD (Avg. Curr.)	Time with Current on (h)	Energy Consumption (J)	Avg. Power (W/m^2^)	Avg. Electrical Resistivity (Ω·cm)	Tenv (Initial) (°C)	Avg. T_Concrete (Initial) (°C)	Avg. T_Concrete (Final) (°C)	ΔT Concrete (°C)	ΔT (Concrete-Env) (°C)	Avg. Powerper °C (W/(m^2^·°C))
L.C.	AC 10	0.5	0.7%	6.1	112,348	71.6	52.8	26.2	24.9	29.3	3.1 ^3^	3.1	23.3
L.C.	DC 10	0.5	2.1%	6.1	114,159	72.6	49.7	26.2	25.1	29.3	3.1 ^3^	3.1	23.7
L.C.	AC 10	1.1	0.3%	5.3	425,633	307.7	49.1	26.1	26.3	37.1	10.8	11.0	27.9
L.C.	DC 20	1.1	0.3%	5.3	436,499	315.7	45.3	26.1	26.2	38.0	11.8	11.9	26.6
L.C.	AC 25	1.4	0.6%	4.7	599,333	489.1	48.2	25.3	25.8	42.3	16.5	17.0	28.8
L.C.	DC 25	1.5	1.0%	4.7	620,458	506.5	44.1	25.3	25.8	44.6	18.8	19.3	26.2
Prev.	AC 20	1.2	0.4%	3.6	313,159	338.7	44.6	−15.0	4.4	4.5	0.1	19.5	17.4
Prev.	DC 20	1.1	0.2%	3.6	286,908	310.3	46.2	−15.0	2.4	2.5	0.2	17.5	17.7
Prev.	AC 25	1.4	0.3%	4.1	513,357	479.3	49.3	−15.0	4.5	7.9	3.5	22.9	20.9
Prev.	DC 25	1.4	0.3%	4.1	503,621	470.2	47.6	−15.0	6.3	9.5	3.2	24.5	19.2
Cur.	AC 20	1.2	0.5%	8.3	711,579	329.4	45.9	−15.0	−13.9	4.3	18.2	19.3	17.0
Cur.	DC 20	1.1	0.4%	8.3	645,834	298.9	48.0	−15.0	−13.6	2.2	15.8	17.2	17.4
Cur.	AC 25	1.5	0.5%	4.7	641,395	526.5	44.7	−15.0	−14.1	11.9	26.0	26.9	19.6
Cur.	DC 25	1.4	0.7%	4.7	595,619	489.2	45.6	−15.0	−13.8	9.4	23.2	24.4	20.0

^1^ L.C.: Laboratory conditions; Prev.: Prevention conditions; Cur.: Curing conditions. ^2^ Average. ^3^ Compared to Tenv, as it is higher than initial T_concrete.
